# Extensive Lymphadenopathy in an HIV‐Negative Patient With Multidrug‐Resistant Tuberculosis

**DOI:** 10.1002/rcr2.70480

**Published:** 2026-01-20

**Authors:** Tomoyuki Araya, Toshiyuki Kita, Kazuhiko Iwasaki, Takayuki Higashi, Ryo Hara, Hazuki Takato

**Affiliations:** ^1^ Department of Respiratory Medicine NHO Kanazawa Medical Center Kanazawa Ishikawa Japan

**Keywords:** EBUS‐TBNA, HIV‐negative, lymphadenopathy, multidrug‐resistant tuberculosis, smear‐negative

## Abstract

Tuberculous lymphadenitis is generally more severe in patients with HIV infection. We present an HIV‐negative patient with multidrug‐resistant tuberculosis who developed severe, extensive lymphadenitis involving multiple extrapulmonary regions. This clinical image highlights that marked lymph node involvement may occur in HIV‐negative patients presenting with pronounced systemic symptoms.

## Graphical abstract

Extensive lymphadenopathy is uncommon in HIV‐negative patients with tuberculosis. This clinical image highlights the striking CT appearance of extensive extrapulmonary lymph node involvement in symptomatic multidrug‐resistant tuberculosis, even in the absence of HIV infection.

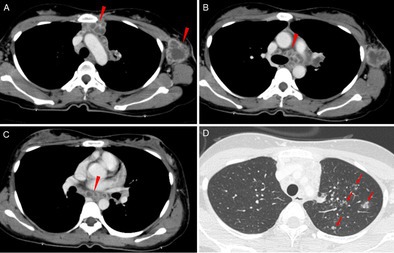

Tuberculous lymphadenitis is often more severe in patients with HIV infection [1]. However, extensive lymphadenopathy has also been reported in HIV‐negative patients with tuberculosis presenting with marked systemic symptoms, such as fever and weight loss [2].

A 26‐year‐old woman presented with a 2‐month history of persistent fever, a 10‐kg weight loss and painful swelling of the left axillary lymph nodes. Contrast‐enhanced chest computed tomography revealed extensive lymphadenopathy involving the mediastinal, hilar and left axillary regions, along with multiple fine nodules in the left upper lobe (Figure 1). Lymph node enlargement was also noted in part of the upper abdomen.

**FIGURE 1:**
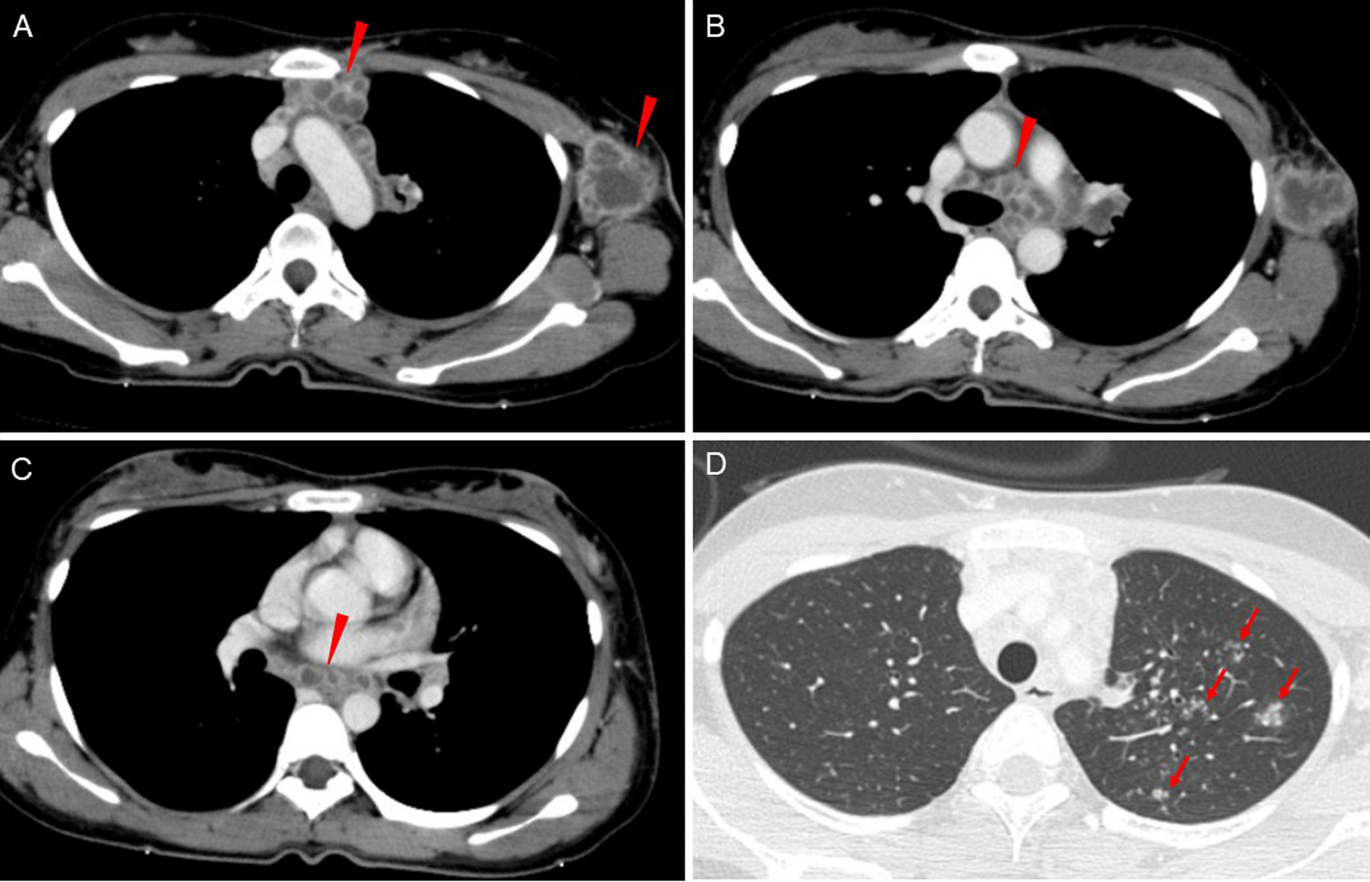
Contrast‐enhanced chest computed tomography on admission. (A) Extensive lymphadenopathy extending from the upper mediastinum to the left axilla, with representative areas showing internal low‐attenuation consistent with necrosis (arrowheads). (B) Marked enlargement of the left lower paratracheal lymph nodes (#4L) with internal low‐attenuation areas consistent with necrosis (arrowhead). (C) Enlarged subcarinal lymph nodes (#7) showing internal low‐attenuation areas consistent with necrosis (arrowhead). (D) Multiple fine nodules in the left upper lobe (Segments S1 + 2), indicated by arrows.

Laboratory testing showed leukocytosis and elevated C‐reactive protein levels. The interferon‐gamma release assay was positive, while HIV antigen/antibody testing was negative. Sputum smears and polymerase chain reaction testing for *Mycobacterium tuberculosis* were negative. Endobronchial ultrasound‐guided transbronchial needle aspiration of mediastinal lymph nodes (#4L and #7) demonstrated granulomas with caseous necrosis (Figure 2), and cultures from lymph node aspirates and sputum grew *Mycobacterium tuberculosis* . Drug susceptibility testing confirmed multidrug‐resistant tuberculosis (MDR‐TB).

**FIGURE 2:**
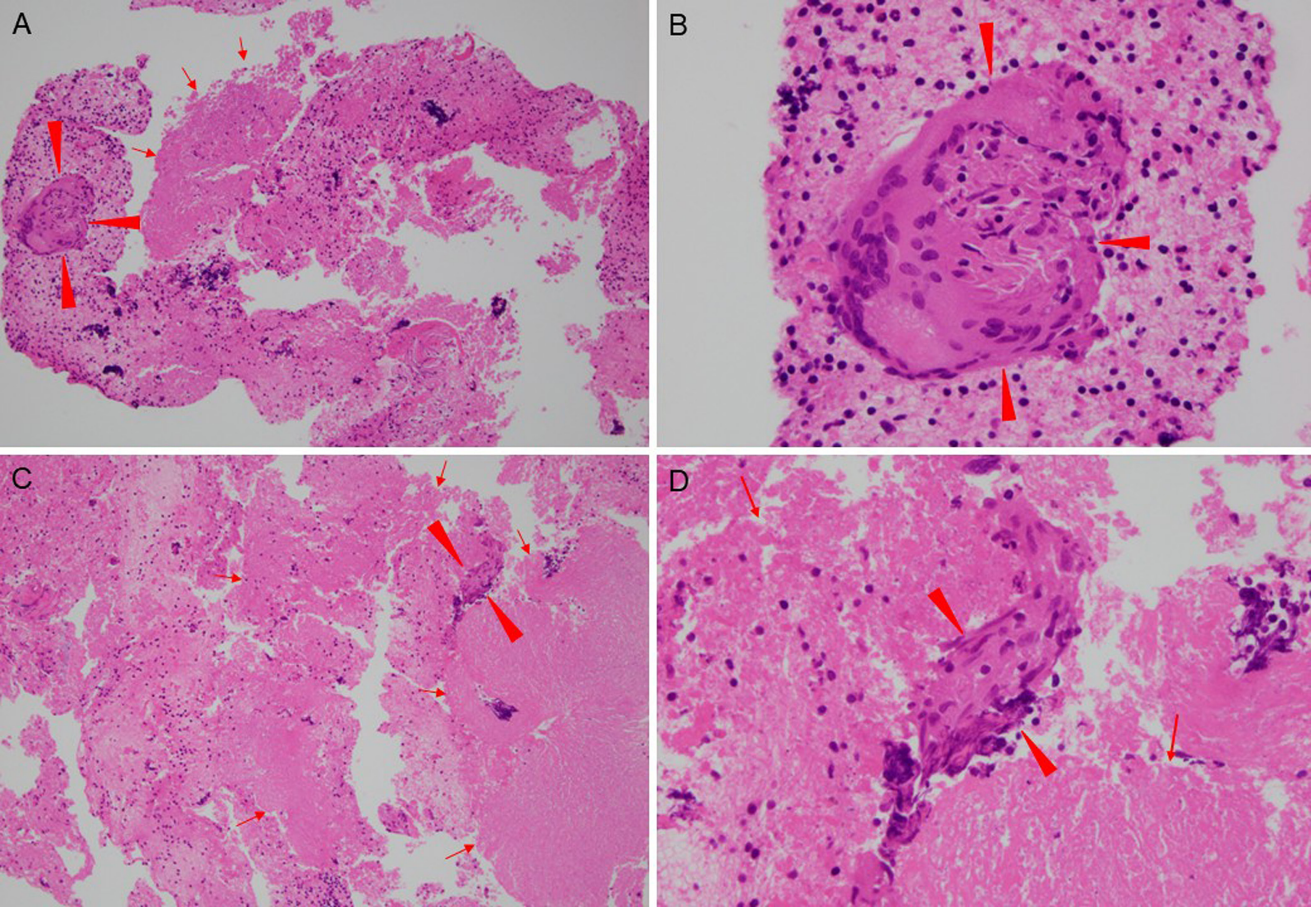
Haematoxylin and eosin–stained sections of lymph node specimens obtained by endobronchial ultrasound‐guided transbronchial needle aspiration (EBUS‐TBNA). (A) A low‐power view (original magnification ×100) of the subcarinal lymph node (#7) demonstrates areas of caseous necrosis (arrows) with fusion of Langhans‐type multinucleated giant cells (arrowheads). (B) A high‐power view (×400) of the corresponding area in panel A shows fusion of Langhans‐type multinucleated giant cells (arrowheads) against a background of fibrin deposition and inflammatory cell infiltration. (C) A low‐power view (×100) of an EBUS‐TBNA specimen from the left lower paratracheal lymph node (#4L) reveals caseous necrosis (arrows) accompanied by fusion of Langhans‐type multinucleated giant cells (arrowheads). (D) A high‐power view (×400) of panel C highlights fusion of Langhans‐type multinucleated giant cells (arrowheads) in the background of caseous necrosis (arrows).

The patient was treated with an 18‐month multidrug regimen including bedaquiline, delamanid and linezolid. This case highlights that MDR‐TB presenting with marked systemic symptoms may be associated with extensive extrapulmonary lymphadenopathy even in HIV‐negative patients.

## Author Contributions

Tomoyuki Araya drafted the manuscript and verified the clinical data. Toshiyuki Kita supervised the work and critically reviewed the manuscript. Kazuhiko Iwasaki, Takayuki Higashi, Ryo Hara and Hazuki Takato reviewed the manuscript and contributed to data verification. All authors read and approved the final manuscript.

## Funding

The authors have nothing to report.

## Ethics Statement

The case report was approved by the ethics committee of the NHO Kanazawa Medical Center (Approval No. R07‐058). The study was conducted ethically in accordance with the World Medical Association Declaration of Helsinki.

## Consent

The authors declare that written informed consent was obtained for the publication of this manuscript and accompanying images using the consent form provided by the Journal.

## Conflicts of Interest

The authors declare no conflicts of interest.

## Data Availability

The data that support the findings of this study are available on request from the corresponding author. The data are not publicly available due to privacy or ethical restrictions.
